# Primary synovial sarcoma of the prostate metastatic to the liver and lung: a case report

**DOI:** 10.1186/1477-7819-12-194

**Published:** 2014-06-27

**Authors:** Qi Zhang, Huiju Wang, Ligang Ren, Xiaolong Qi, Feng Liu, Dahong Zhang

**Affiliations:** 1 Department of Urology, Zhejiang Provincial People’s Hospital, Hangzhou, China; 2 Key Laboratory of Gastroenterology of Zhejiang Province, Hangzhou, China

**Keywords:** Prostate, Synovial sarcoma, Metastasis

## Abstract

Primary synovial sarcoma of the prostate is an uncommon malignant tumor. There are few cases reported in the English medical literature to date. Here, we present a case of 22-year-old man with primary synovial sarcoma of the prostate metastatic to the liver and lung. To our knowledge, only six reports of synovial sarcoma involving the prostate have been previously published. We also reviewed the previous treatments and prognoses in previous case reports and evaluate the proper treatment for this disease.

## Background

Primary synovial sarcomas have been described at unusual sites, including the heart, oesophagus, larynx, pleura, lung, kidney, prostate, liver, abdominal wall, retroperitoneum and gastrointestinal tract; involvement of the genitourinary tract is exceedingly rare [1-3]. Synovial sarcoma arising primarily from the prostate is a rare occurrence, with only six previously reported cases. Synovial sarcoma of the prostate is usually identified at a late stage because of its nonspecific presentation. The purpose of our article is to add an additional case to the literature, to review the literature, and to formulate treatment recommendations. We describe a patient presenting with primary synovial sarcoma of the prostate metastatic to the liver and lung.

## Case presentation

**Figure 1:**
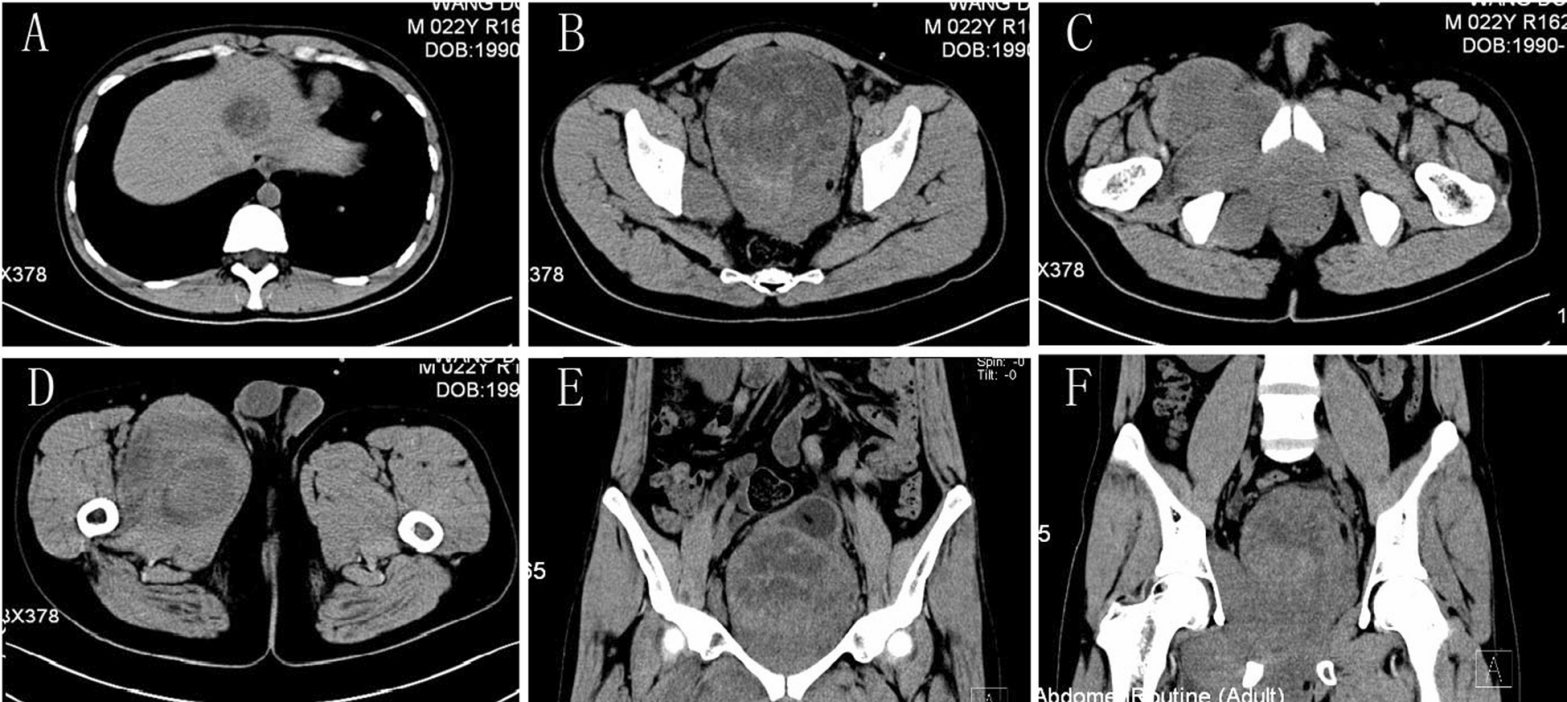
**Enhanced pelvic computed tomography. (A)** Evidence of liver metastasis. **(B-F)**, Enhanced pelvic computed tomography revealed a 14-cm mass that appeared to originate in the prostatic fascia. An 8.5-cm mass was found on pelvic magnetic resonance imaging.

**Figure 2:**
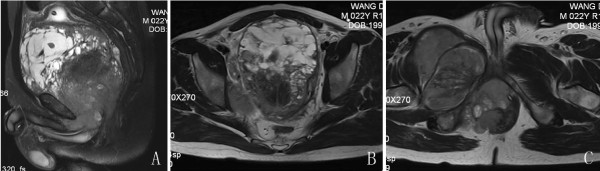
**Enhanced pelvic magnetic resonance imaging. (A-C)** Enhanced pelvic T2 weighted MR revealed a high signal mass originating in the prostatic fascia and an 8.5-cm mass was found in the right groin area.

**Figure 3:**
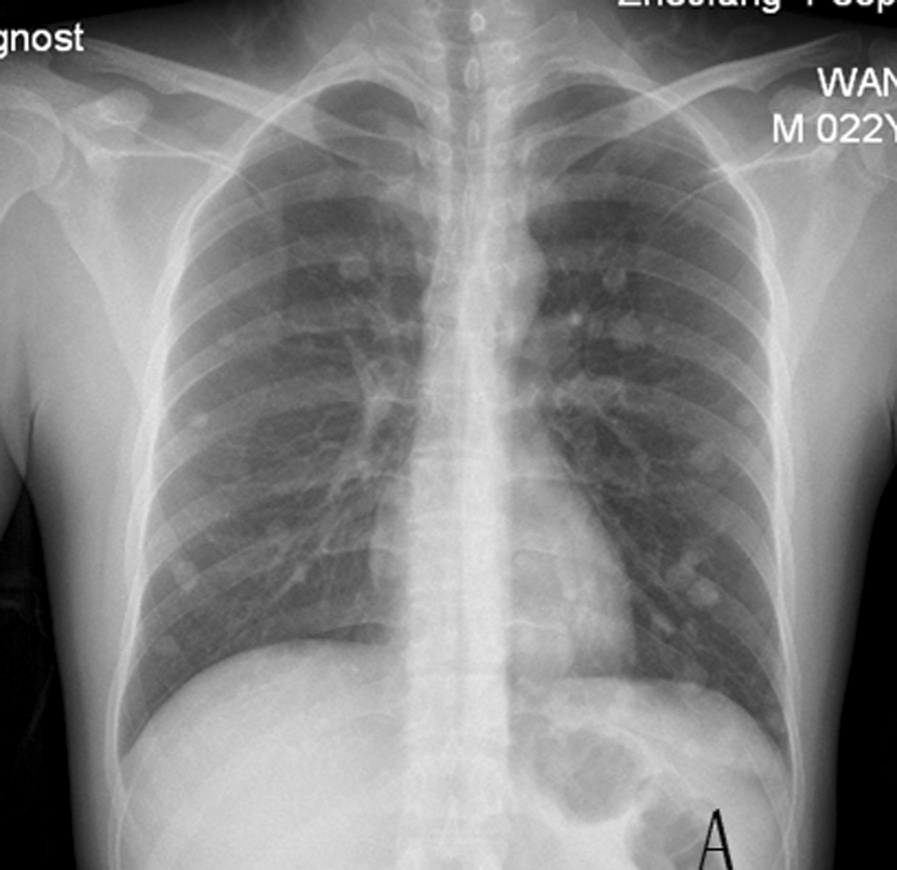
**Chest radiography. (A)** Evidence of lung metastasis.

**Figure 4:**
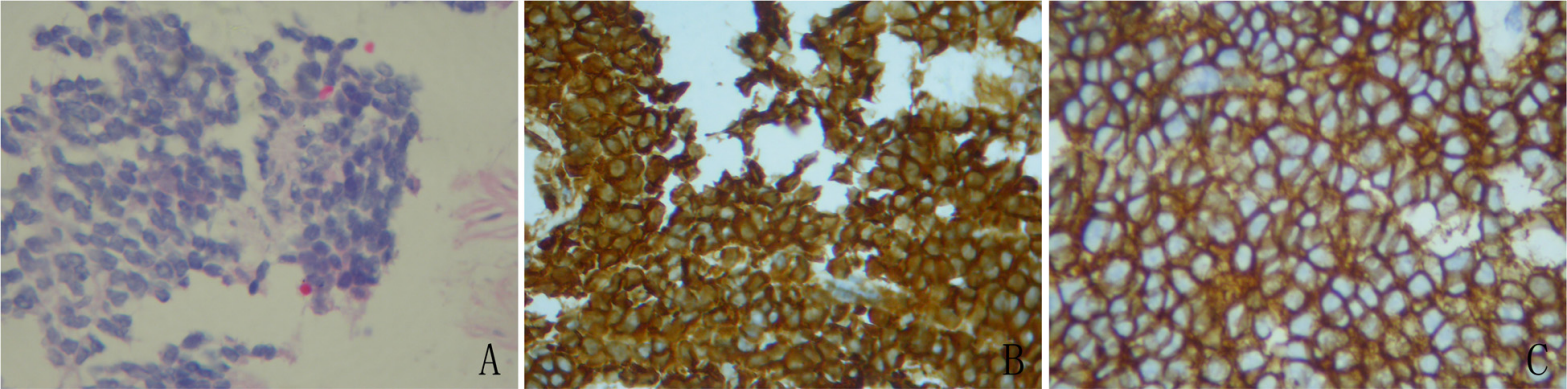
**Pathologic analysis. (A)** The pathologic findings of the tumor (H&E). **(B)** Immunohistochemical staining for vimentin in tumor tissue. **(C)** Immunohistochemical staining for CD99 in tumor tissue.

A 22-year-old man presented elsewhere with lower urinary tract symptoms and was referred to us for further evaluation because of urinary retention. No particular family history or occupational hazard was elicited. Our patient complained of dysuria, urinary frequency and nocturia five to ten times per night before urinary retention. On digital rectal examination, a large prostatic mass was palpable, and the surface was smooth. His serum prostate-specific antigen was 1.2 ng/ml (normal 4 or less). Pelvic computerized tomography (CT) imaging (Figure 1) and magnetic resonance imaging (MRI) (Figure 2) demonstrated a 14-cm mass that appeared to originate in the prostatic fascia. Another 8.5-cm mass was found in the right groin area. CT and chest radiography showed evidence of liver and lung metastasis (Figure 3). The patient’s age was younger than the common age range of patients for the commonest types of prostatic adenocarcinoma. Ultrasound-guided transrectal needle biopsy and pathologic examination revealed synovial sarcoma. The pathologic findings were consistent with the diagnosis of synovial sarcoma arising from the prostate (Figure 4). On immunohistochemical staining, the tumor cells were positive for vimentin and CD99, but negative for alpha smooth muscle actin, desmin, and S-100 protein. The presence of SYT-SSX fusion transcript was confirmed by RT-PCR using genomic DNA isolated from paraffin blocks. The patient refused to receive any therapeutic schedule including irradiation、chemotherapy and radical prostatectomy. His disease progression was rapid. At three months of follow-up, the patient had developed multiple lung metastases and subsequently died of respiratory failure.

## Discussion

Synovial sarcoma is a clinically and histomorphologically well-defined soft tissue tumor that is extremely uncommon, with about 90% synovial sarcomas occurring on the extremities. In most patients with this disease, distant pulmonary metastases develop after primary tumor surgery. In the urinary system, most synovial sarcomas have been reported in the kidney [4]. Primary synovial sarcomas involving the prostate are exceedingly rare, with only a handful of cases reported in the English medical literature to date (Table 1) [5,6].

**Table 1:** Clinicopathologic features, treatment, and prognosis of seven cases of primary synovial sarcoma of prostate

	**Cases**						
	**Iwasaki**	**Shirakawa**	**Williams**	**Pan**	**Li first case**	**Li second case**	**This study**
Age (years)	37	52	63	44	46	44	22
Clinical findings	Gross hematuria, dysuria, painful micturition	Urinary retention	Lower urinary tract symptoms	Lower urinary tract symptoms	Dysuria, painful micturition	Dysuria gradually	Dysuria, urinary frequency, nocturia, urinary retention
Serum PSA (ng/ml)	None	0.9	0.5	2.91	0.35	1.19	1.2
Extent of tumor	10-cm solid mass with necrosis, invading the prostatic urethra, seminal vesicles, retrovesical soft tissues	7-cm mass, originating in the prostatic fascia of the right lobe	8.5-cm mass extending from the mid-potion of the prostate inferiorly to the base of the penis	6-cm, well circumscribed, soft, heterogeneous mass in the right side of prostate	5.5-cm mass with partial necrosis extending to the pelvic soft tissue	12-cm mass extending to the bladder, the rectum and pelvic soft tissue, partial necrosis	14-cm mass that appeared to originate in the prostatic fascia. Another 8.5-cm mass was found in the right groin area
IHC results	VM+, EMA +	VM+	VM+, CK(CAM5.2)+, S100+, CK7+, CKAE1/3+,CAL+	VM+, CD99+, CK +, bcl-2+	VM+, CD99+, bcl-2+, E-CA+, CK/EMA +	VM+, CK+,CD99+, E-CA+, bcl-2+	VM+, CD99+
Treatment	Chemotherapy, radical prostatocystectomy	Radical prostatectomy, adjuvant chemotherapy	Irradiation with *en bloc* penectomy and pubectomy and ileal conduit urinary diversion	Radical prostatectomy	Radical prostatectomy, chemotherapy	The tumor could not be completely resected	None
Prognosis	Died (32 months, tumor recurrence and metastasis to the lungs and liver)	Alive (no recurrence or metastasis)	Alive (no recurrence or metastasis)	Alive (no recurrence and metastasis)	Alive (no recurrence and metastasis)	Died (eight months, tumor recurrence and metastasis to the costosternal junction and lung)	Died (three months, multiple lung metastasis and respiratory failure

Primary prostatic sarcomas are rare tumors and most patients present with urinary obstruction, as did our patient. The pathologic findings were consistent with the diagnosis of synovial sarcoma arising from the prostate. In common with other synovial sarcomas, the immunohistochemical staining demonstrated some typical findings of synovial sarcoma: the tumor cells were positive for vimentin and CD99, but negative for CD34, Bcl-2, alpha smooth muscle actin, desmin, and S-100 protein. Finally, the presence of SYT-SSX fusion transcript confirmed the diagnosis [7,8].

Several clinicopathologic features were particular for primary synovial sarcoma of the prostate. As compared with the most common malignant tumor of the prostate adenocarcinoma, prostatic sarcoma occurs in relatively younger patients [9]. According to the previous reports, the age of diagnosis for these patients was 37 to 63 years. The age of our patient (22 years old) was younger than this range. Most patients with prostatic sarcoma present with symptoms of bladder outlet obstruction. Our patient complained of dysuria, urinary frequency and nocturia before urinary retention. The massive tumor may have been responsible for this symptom. The PSA level of prostatic adenocarcinoma is often elevated. It has been reported that the PSA level may not rise in patients with prostatic sarcoma, due to the non-epithelial origin of prostate sarcoma [9]. PSA levels in previous patients with prostatic synovial sarcoma ranged from 0.5 ng/ml to 0.9 ng/ml. Our patient had a PSA level of 1.20 ng/ml, which was still within normal range. Prostatic synovial sarcoma image findings are not well reported. In one case report by Shirakawa, T2 weighted MRI revealed a high signal mass originating in the prostatic fascia [5]. The imaging findings of primary synovial sarcoma of the prostate are not unique and pathologic examination is the only way to make a definite diagnosis.

The survival of persons with primary prostatic sarcomas is poor, with most patients dying within a few months after diagnosis and surgery. Distant pulmonary metastases are found in most patients with this disease, whether the patient has undergone surgery or not. In our case, the tumor had metastasis to the liver and lung. Treatment for this sarcoma is uncertain due to extremely limited experience but it appears that aggressive resection has to be considered for the treatment of locally confined synovial sarcoma [10]. The role of adjuvant chemotherapy and/or radiation remains unknown; however, it is strongly recommended because of the poor prognosis. Documentation of further cases is needed to establish appropriate therapy.

## Conclusion

In conclusion, we demonstrate a case of primary prostatic synovial sarcoma. Due to the paucity of cases of synovial sarcoma involving the prostate, protocols for definitive treatment are not well defined. Treatment for this tumor is uncertain due to extremely limited experience, but it appears that aggressive resection should be considered as the standard therapy. The role of adjuvant chemotherapy and radiation also remains unknown. Reporting of future cases is necessary to establish appropriate therapeutic guidelines for this disease.

## Consent

Written informed consent was obtained from the patient for publication of this case report and accompanying images. A copy of the written consent is available for review by the Editor-in-Chief of this journal.

## Abbreviations

CT: computerized tomography; MRI: magnetic resonance imaging; RT-PCR: reverse transcription-polymerase chain reaction; DNA: Desoxvribose Nucleic Acid.

## Competing interests

The authors declare that they have no competing interests.

## Authors’ contributions

QZ, LR, HW, XQ, FL and DZ participated in the admission and the care of this patient. All the authors participated in the conception, the design, data collection and interpretation, manuscript preparation and literature search. All authors have read and approved the final manuscript.
